# Predictive factors of submucosal fibrosis before endoscopic submucosal dissection for superficial squamous esophageal neoplasia

**DOI:** 10.1038/s41424-018-0024-5

**Published:** 2018-06-15

**Authors:** Cheal Wung Huh, Han Hee Lee, Byung-Wook Kim, Joon Sung Kim, Bo-In Lee, Chul-Hyun Lim, Jiyoung Kim

**Affiliations:** 1 Division of Gastroenterology, Department of Internal Medicine, Incheon St. Mary’s Hospital, College of Medicine, The Catholic University of Korea, Incheon, Korea; 2 Division of Gastroenterology, Department of Internal Medicine, Seoul St. Mary’s Hospital, College of Medicine, The Catholic University of Korea, Seoul, Korea; 3 Department of Pathology, Incheon St. Mary’s Hospital, College of Medicine, The Catholic University of Korea, Incheon, Korea

## Abstract

**Objectives:**

Endoscopic submucosal dissection (ESD) is an effective treatment modality for superficial squamous esophageal neoplasia (SSEN). However, submucosal fibrosis is an important obstacle to successful ESD. The aim of this study was to determine the ESD outcome in relationship to the degree of submucosal fibrosis of SSEN and to identify factors for predicting submucosal fibrosis.

**Methods:**

We retrospectively analyzed endoscopic and pathologic factors related to submucosal fibrosis in 41 patients with SSEN who underwent ESD. Also, *en bloc* resection rate, complication rate, and procedure time according to the degree of submucosal fibrosis were evaluated. Masson’s trichrome staining was used to evaluate histologic submucosal fibrosis.

**Results:**

A depressed type tumor (vs. nondepressed type tumor, *P* = 0.002), length of tumor greater than 20 mm (vs. length of tumor ≤ 20 mm, *P* = 0.036), and delayed ESD after initial biopsy (vs. immediate ESD after initial biopsy, *P* = 0.005) were independent factors predictive of submucosal fibrosis. The severity of submucosal fibrosis was significantly associated with a higher complication rate such as bleeding and perforation. Also, as the severity of the submucosal fibrosis increased, the amount of time required for the ESD procedure increased.

**Conclusions:**

Length of tumor greater than 20 mm and endoscopic depressed type are endoscopic predictive factors of submucosal fibrosis in SSEN. Moreover, to avoid submucosal fibrosis, ESD should be attempted immediately after biopsy for the diagnosis of SSEN.

## Introduction

Endoscopic submucosal dissection (ESD) is considered a feasible procedure for superficial squamous esophageal neoplasia (SSEN)^1–4^. Compared with endoscopic mucosal resection (EMR), ESD has many advantages such as a higher *en bloc* resection rate and a lower rate of recurrence^5–8^. However, esophageal ESD is a more difficult procedure to perform than esophageal EMR. Also, esophageal ESD is technically more difficult than gastric ESD. Therefore, esophageal ESD is more likely to lead to a higher frequency of complications (e.g., bleeding and perforation) compared with esophageal EMR or gastric ESD.

Success rate of ESD depends on the expertize of the endoscopist, features of the neoplasia and various applied techniques^7, 9^. Lesion factors, such as submucosal fibrosis, are important obstacles to success. Previous studies demonstrated that there was significant association between the degree of submucosal fibrosis and the outcome of ESD in gastric and colorectal neoplasia^10–12^. To our knowledge, however, there has been no report on the relationship between the degree of submucosal fibrosis and the outcome of ESD in SSEN. In addition, the prediction of submucosal fibrosis prior to ESD might be helpful for successful procedure. Therefore, we retrospectively investigated the following factors: (1) the association between clinicopathological factors and endoscopic submucosal fibrosis, (2) the relationship between the degree of endoscopic submucsoal fibrosis and the outcome of ESD, (3) the agreement between endoscopically-observed submucsoal fibrosis and pathologically-observed submucosal fibrosis.

## Methods

### Patients

Between January 2011 and December 2016, a total of 63 patients were diagnosed as esophageal tumors and underwent ESD at Incheon St. Mary’s Hospital and at Seoul St. Mary’s Hospital, The Catholic University Korea. Twenty two patients with a subepithelial lesion (e.g., gastrointestinal stromal tumor, leiomyoma, granular cell tumor) were excluded and 41 patients were selected for retrospective analysis. The Institutional Review Boards (IRBs) of The Catholic University of Korea approved this study (OC17RESI0131).

### ESD procedures

All ESD procedures were performed by three expert ESD endoscopists (B.W.K., B.I.L., J.S.K). Patients were moderately sedated with midazolam and propofol, while the ESD was performed. A video endoscope with a water-jet function (GIF-HQ290, GIF-Q260J; Olympus, Tokyo, Japan) was used. A disposable distal transparent cap (D-201-11804; Olympus, Tokyo, Japan) was mounted on the tip of the endoscope in all cases. To identify the target lesion, chromoendoscopy with Lugol’s solution or narrow band imaging with magnification was used. The area around the lesion was marked with argon plasma coagulation. A mixture of 10% glycerol solution and diluted epinephrine (1:200,000) was injected into the submucosal layer under the lesion. Epinephrine (1:1000, total epinephrine 1 mg) were mixed in a 200-mL container of Glycerol, and 8 mL of the solution was drawn into 10-mL disposable syringe to use for SSEN. Carbon dioxide was used for the insufflation. The ESD procedure was performed mainly with a dual knife (KD-650Q; Olympus,Tokyo, Japan) or with an IT-knife 2 (KD-610L; Olympus, Tokyo, Japan) or with hook knife (KD-620LR; Olympus, Tokyo, Japan). Hemostatic forceps (Coagrasper, FD-410LR; Olympus, Tokyo, Japan) with a soft coagulation mode were used to control bleeding during the procedure.

### Definitions

**Fig. 1:**
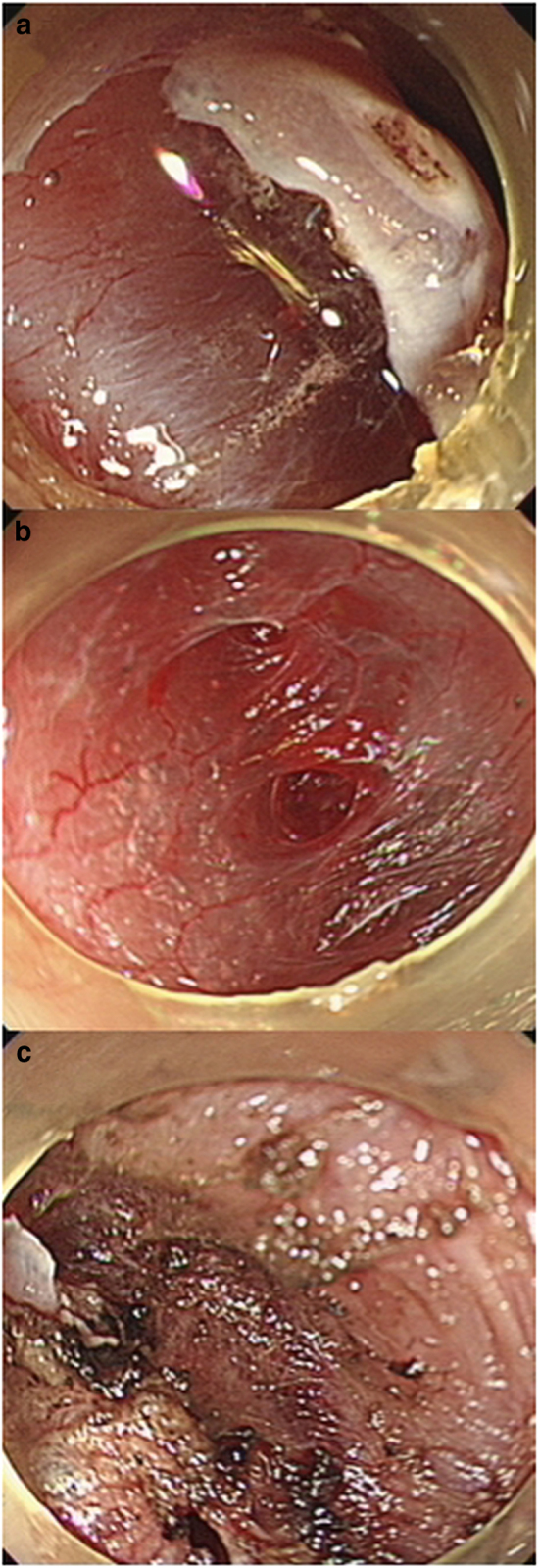
Degree of fibrosis in the submucosal layers in superficial squamous esophageal neoplasia. **a** F0, no fibrosis, which manifested as a blue transparent layer. **b** F1, mild fibrosis, which appears as a white web-like structure in the blue submucosal layer. **c** F2, severe fibrosis, which appears as a white muscular structure without a blue transparent layer in the submucosal layer

Endoscopically, the degree of submucosal fibrosis was categorized as follows based on the observation at the time of the injection of glycerol mixture (Fig. 1); F0, no fibrosis, which manifests as a transparent layer; F1, mild fibrosis, which appeared as a white web-like structure in the submucosal layer; and F2, severe fibrosis, which appeared as a white muscular structure without a transparent layer in the submucosal layer^12^.

**Fig. 2:**
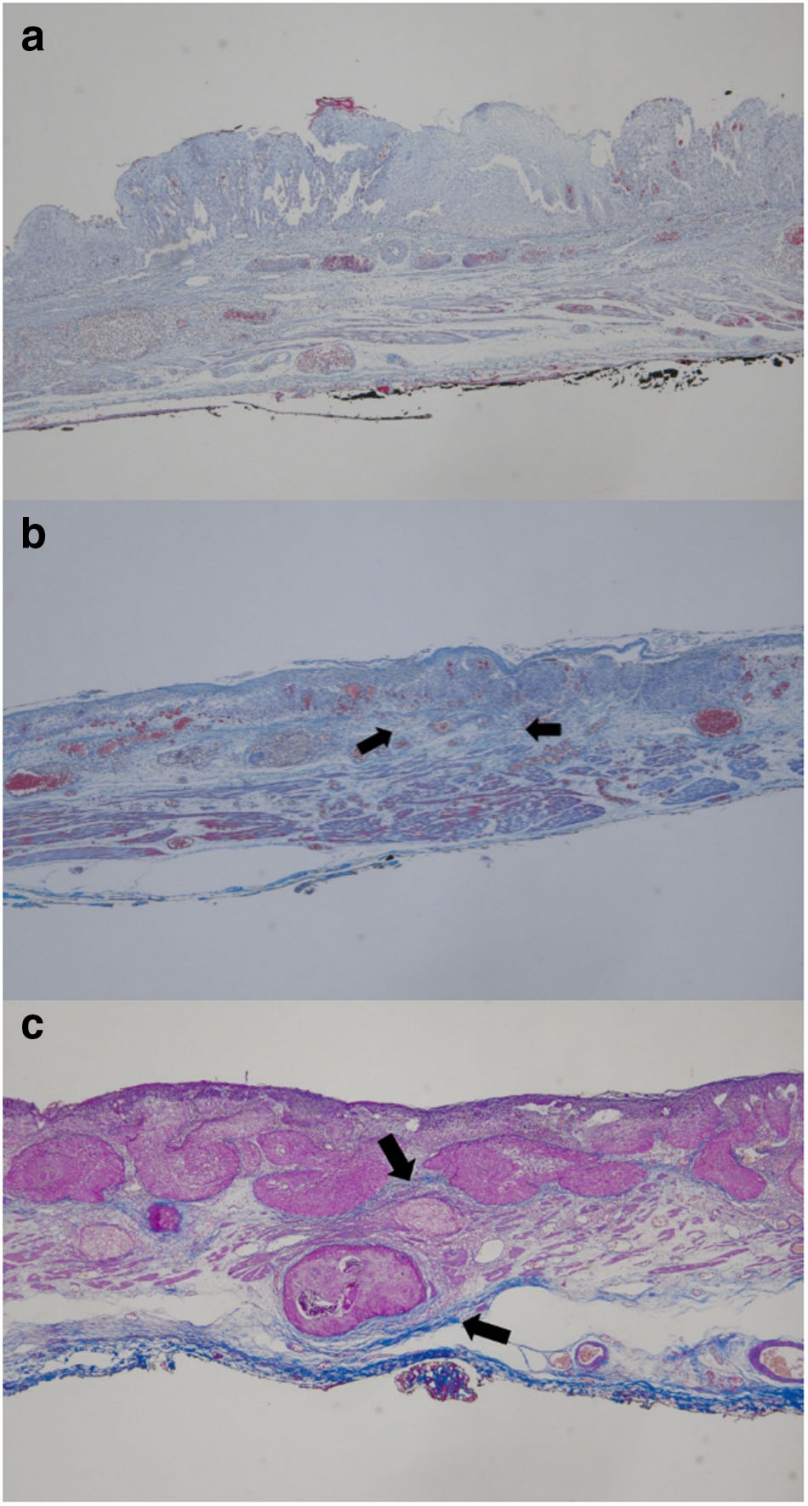
Histologic assessment of the submucosal fibrosis using Masson’s trichrome stain. (Blue: collagen (arrows), Pink: cytoplasm; Brown-dark blue: nuclei) **a** F0, no fibrosis. (intensity, 0; extent, 0; sum, 0) **b** F1, mild fibrosis. (intensity, 1; extent, 1; sum 2) **c** F2, severe fibrosis. (intensity, 2; extent, 2; sum, 4). (Masson’s trichrome stain, × 40)

The histological sections were examined by an expert pathologist (J.K.) who was blinded to all clinical information. Masson’s trichrome staining was used to evaluate submucosal fibrosis. The intensity of the submucosal fibrosis was evaluated as follows; 0, negative stain, no fibrosis, nearly normal appearance; 1, weak fibrosis; and 2, dense fibrosis. The extent of fibrosis was calculated by measuring the percentage of the total area stained with Masson’s trichrome stain, as follows; 0, 0–10%; 1, 11–50%; and 2, 51–100%. The staining score was finally collected as the sum of the intensity and extent scores. A final score of 0 with fibrosis was considered as no fibrosis (F0), 1 and 2 as mild fibrosis (F1), and 3 and 4 as severe fibrosis (F2), respectively (Fig. 2).

Perforation was defined as a hole which is visible in the esophageal wall where the mediastinal cavity is exposed. Immediate bleeding was defined as spurting or oozing of blood from a vessel during the ESD procedure. Delayed bleeding was defined as bleeding with hematemesis or melena that required endoscopic re-intervention or transfusion after the ESD procedure. The procedure time was defined as the time from the insertion of the endoscope to complete resection of the lesion. The endoscopic gross appearances of SSEN were classified according to the Paris classification as protruding (I), non-protruding and non-excavated (II), or excavated (III)^13^. Type II lesions were subclassified as slightly elevated (IIa), flat (IIb), or slightly depressed (IIc). Next, all lesions were broadly classified into two groups: depressed type (IIc, III) and nondepressed type (I, IIa, IIb).

### Statistical analysis

Chi-square tests and Fisher’s exact tests were used to evaluate associations among the various categorical variables, and *t*-tests were used for non-categorical variables in the intergroup comparisons of clinicopathologic characteristics. A multiple logistic regression analysis was used to determine independent predictors of submucosal fibrosis. In addition, a kappa statistic was used to measure the agreement between the estimates of the endoscopic submucosal fibrosis and histologic submucosal fibrosis. The accepted significance level was a *P* value less than 0.05. All statistical analyses were performed using the SPSS version 18.0 for Windows software (SPSS Inc., Chicago, IL, USA).

## Results

### The relationship between clinicopathologic factors and endoscopic submucosal fibrosis

Clinicopathologic factors associated with endoscopic submucosal fibrosis are displayed in Table 1. A univariate analysis demonstrated that endoscopic submucosal fibrosis was significantly associated with length of tumor, endoscopic gross appearance, and timing of ESD from initial biopsy. In particular, severe submucosal fibrosis (F2) was closely related to larger tumor size (>20 mm) and delayed ESD after initial biopsy. The multivariate logistic regression analysis indicated that a depressed type tumor (vs. nondepressed type tumor, *P* = 0.002), length of tumor greater than 20 mm (vs. length of tumor ≤20 mm, *P* = 0.036), and delayed ESD after initial biopsy (vs. early ESD after initial biopsy, *P* = 0.005) were independent factors that predicted endoscopic submucosal fibrosis (Table 2).

**Table 1:** Univariate analysis of the clinicopathological factors associated with submucosal fibrosis

**Variables**	**Submucosal fibrosis (** ***n*** **, %)**			***P*** **value**
	**No**	**Yes**		
	F0 (*N* = 21)(*n*, %)	F1 (Mild) (*N* = 15) (*n*, %)	F2 (Severe) (*N* = 5) (*n*, %)	
Sex				0.095
Male	16 (80.0)	13 (86.7)	3 (60.0)	
Female	4 (20.0)	2 (13.3)	2 (40.0)	
Age (yr)	63.7 ± 8.7	65.1 ± 10.1	67.0 ± 8.8	0.785
Tumor location				0.252
Upper	0 (0)	1 (6.7)	1 (20.0)	
Middle	14 (66.7)	9 (60.0)	4 (80.0)	
Lower	7 (33.3)	5 (33.3)	0 (0)	
Length of the tumor (mm)				0.013
≤20	15 (71.4)	7 (46.7)	0 (0)	
>20	6 (28.6)	8 (53.3)	5 (100)	
Circumferential extension				0.100
<1/4	7 (33.3)	5 (33.3)	0 (0)	
1/4 – 2/4	12 (57.2)	5 (33.3)	2 (40.0)	
2/4 – 3/4	2 (9.5)	5 (33.3)	3 (60.0)	
≥3/4	0 (0.0)	0 (0.0)	0 (0)	
Endoscopic gross appearance				0.047
Depressed	0 (0)	4 (26.7)	1 (20.0)	
Non-depressed	21 (100.0)	11 (73.3)	4 (80.0)	
Ulceration				0.411
Yes	0 (0)	1 (6.7)	0 (0)	
No	21 (100.0)	14 (93.3)	5 (100.0)	
Time from diagnosis to ESD (days)				0.003
≤21	15 (71.4)	4 (26.7)	0 (0)	
>21	6 (28.6)	11 (73.3)	5 (100.0)	
Histologic findings				0.367
Dysplasia	2 (9.5)	0 (0)	0 (0)	
Squamous carcinoma	19 (90.5)	15 (100.0)	5 (100.0)	
Depth of invasion				0.090
Mucosa	18 (85.7)	10 (66.7)	2 (40.0)	
Submucosa	3 (14.3)	5 (33.3)	3 (60.0)	
Lymphovascular invasion				0.583
Absence	18 (94.7)	15 (100.0)	5 (100.0)	
Presence	1 (5.3)	0 (0)	0 (0)	

*ESD* endoscopic submucosal dissection

**Table 2:** Multivariate logistic regression analysis of factors predicting submucosal fibrosis

**Variables**		**HR (95% CI)**	***P*** **value**
Endoscopic gross appearance	Depressed	12.820 (2.506 – 65.456)	0.002
	Non-depressed	Ref	
Length of the tumor (mm)	>20	6.056 (1.129 – 32.492)	0.036
	≤20	Ref	
Timing of ESD from initial biopsy (days)	>21	10.214 (2.021 – 51.630)	0.005
	≤21	Ref	

*HR* hazard ratio, *ESD* endoscopic submucosal dissection

### Outcome of ESD according to endoscopic submucosal fibrosis

The overall *en bloc* resection rate was not different between the lesions accompanied by endoscopic submucosal fibrosis and without endoscopic submucosal fibrosis. However, the severity of endoscopic submucosal fibrosis was significantly associated with higher immediate bleeding rate. Also, one case of perforation displayed an endoscopic submucosal fibrosis of F2. On the other hand, delayed bleeding was not related to the degree of the endoscopic submucosal fibrosis. (Table 3)

**Table 3:** Immediate bleeding, delayed bleeding, and perforation according to the degree of endoscopic submucosal fibrosis

**Variables**	**Endoscopic submucosal fibrosis**			***P*** **value**
	**No (F0)**	**Mild (F1)**	**Severe (F2)**	
Immediate bleeding				0.018
Yes (*n* = 34)	14 (66.7)	15 (100.0)	5 (100.0)	
No (*n* = 7)	7 (33.3)	0 (0.0)	0 (0.0)	
Delayed bleeding				0.284
Yes (*n* = 3)	2 (9.5)	0 (0.0)	1 (20.0)	
No (*n* = 38)	19 (90.5)	15 (100.0)	4 (80.0)	
Perforation				0.025
Yes (*n* = 1)	0 (0.0)	0 (0.0)	1 (20.0)	
No (*n* = 40)	21 (100.0)	15 (100.0)	4 (80.0)	

**Fig. 3:**
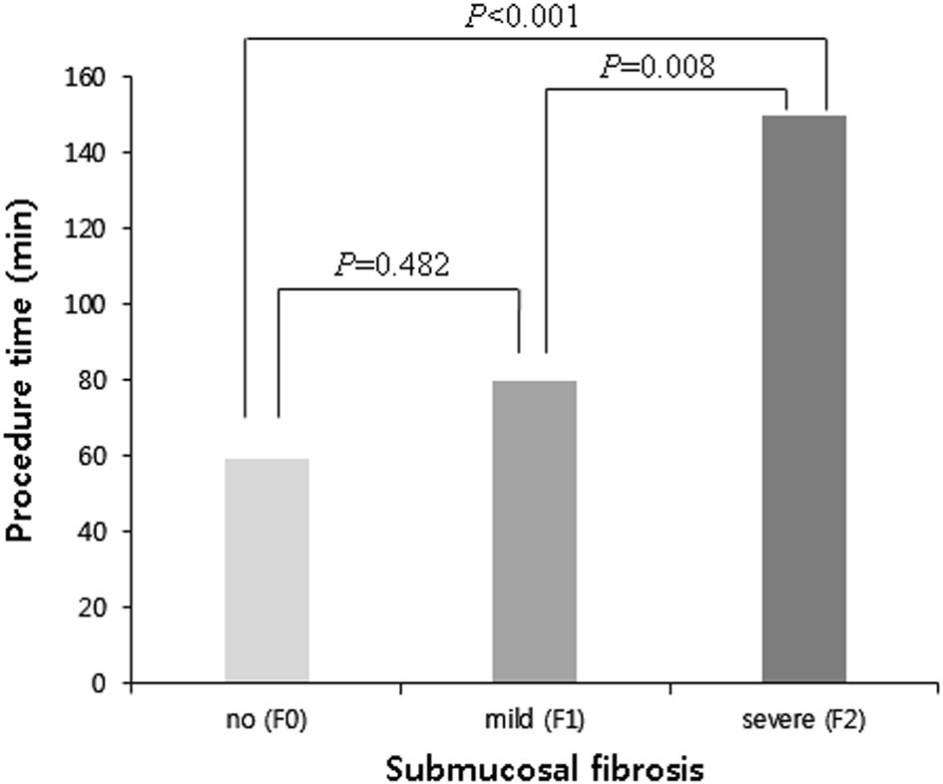
Relationship between procedure times and degree of endoscopic submucosal fibrosis. F0, no fibrosis; F1, mild fibrosis; F2, severe fibrosis

The mean procedure time was 77.9 min. Procedure time was correlated to the degree of endoscopic submucosal fibrosis (Fig. 3). The mean procedure time of the ESD according to the degree of the endosopic submucosal fibrosis were as follows: F0, 59.4 min (ranges 43.6–75.1 min), F1, 79.8 minutes (ranges 57.4–102.2 min), and F2, 150.0 min (ranges 60.7–239.3 min). Obviously, as the endoscopic submucosal fibrosis became more severe, the required procedure time increased.

### Relationship between endoscopic and histologic estimates of submucosal fibrosis

The comparison of endoscopic and histologic grading of submucosal fibrosis is provided in Table 4. The concordance was 87.9% (Kendall’s tau-b and *k* coefficients were 0.877 and 0.808, respectively) (*P* *<* 0.001).

**Table 4:** Degree of endoscopic submucosal fibrosis in relationship to histologic submucosal fibrosis

**Endoscopic submucosal fibrosis**	**Histologic submucosal fibrosis**		
	**No (F0)**	**Mild (F1)**	**Severe (F2)**
No (F0)	11	2	0
Mild (F1)	0	13	2
Severe (F2)	0	0	5

*К* *=* 0.808 (*p* < 0.001)

## Discussion

In this study, three predictive factors of endoscopic submucosal fibrosis were identified: depressed type tumor, length of tumor greater than 20 mm, and delayed ESD after initial biopsy. These findings indicate that technical difficulty will be encountered during esophageal ESD for such lesions. This study also demonstrated that the extent of the endoscopic submucosal fibrosis is related to the frequency of immediate bleeding and endoscopic submucosal fibrosis was classified as F2 in a case with perforation. In addition, as the endoscopic submucosal fibrosis became more severe, the procedure time took longer.

It has been reported that as the size of lesions increased, the dangers of bleeding, perforation, incomplete resection, and delays in procedure increased^14–17^. Furthermore, some studies reported that endoscopic submucosal fibrosis was closely related to the size of tumor and endoscopic gross appearance^10, 11^. However, there have been no examinations of the relationship between the degree of submucosal fibrosis and cliniopathological factors in SSEN. Similar to previous studies on gastric tumors, the present study demonstrated that a length of tumor greater than 20 mm and endoscopic depressed type were independent risk factors for endoscopic submucosal fibrosis. Therefore, we suggest that prior to perform esophageal ESD, a cautious approach is necessary when the length of tumor is over 20 mm and depressed endoscopically since the possibility of endoscopic submucosal fibrosis is very high.

The optimal timing from initial biopsy to ESD remains undefined. A previous study suggested an endoscopic resection should be performed within 21 days after a biopsy because interval over this period may be associated with non-lifting signs in endoscopically-resectable colorectal cancer^18^. Our results also demonstrated that delayed ESD over 21 days after initial biopsy was a significant risk factor of submucosal fibrosis, which suggests that a longer interval between diagnostic biopsies and ESD may lead to submucosal fibrosis. It is plausible that a biopsy can induce mucosal ulceration and cause scarring to change over time, which in turn might lead to submucosal fibrosis. Immediate ESD within 21 days after initial diagnostic biopsy should be considered before submucosal fibrosis proceeds.

We observed that the more severe the endoscopic submucosal fibrosis is, the longer the procedure time takes. Several previous studies also have shown that submucosal fibrosis could be recognized as a predictor of prolonged ESD^11, 14, 17^. In addition, our study revealed that the severity of the endoscopic submucosal fibrosis is associated with the frequency of immediate bleeding and perforation. The prolonged duration of the procedure and high frequency of complications are due to the technical difficulty of dissecting deeper tissue for complete resection where fibrosis occurs. Therefore, when submucosal fibrosis is detected, it is necessary to perform ESD more carefully.

We tested the concordance between the endoscopic and histologic classifications of submucosal fibrosis. The endoscopic classification directly reflects the histological classification of submucosal fibrosis. This suggests that the endoscopic classification could predict the ESD outcome with a high degree of concordance. The endoscopic assessment of submucosal fibrosis is an objective measure of submucosal fibrosis.

Our study has some limitations. First, this study was based on a small sample size. Though not statistically significant, our results showed that endoscopic submucosal fibrosis has the tendency to be associated with the depth of invasion (*p* = 0.063). Further studies with large sample size might lead to statistical significance of the values between the groups. Second, because there is no objective definition of endoscopic submucosal fibrosis and histologic submucosal fibrosis, a subjective classification of a submucosal fibrosis from the observer’s point of view may have caused a difference from other previous studies. To overcome this limitation, we tested the concordance between the endoscopic and histologic classifications of submucosal fibrosis, which demonstrated strong agreement (*κ* = 0.808, *p* < 0.001). Third, there might be a bias due to the retrospective nature of the current study. Finally, additional endoscopic predictive factors, including the expertize of the endoscopist and the method of ESD were not included in this study. Thus, further large prospective studies are needed to validate our study. To our knowledge, however, our study is the first to describe the relationship between the degree of endoscopic submucosal fibrosis and the outcome of ESD in SSEN. In addition, the prediction of endoscopic submucsoal fibrosis prior to ESD would be beneficial for safe and complete endoscopic treatment of SSEN. Therefore, our study provides an insight into the validation of the predictors of endoscopic submucosal fibrosis prior to esophageal ESD.

In conclusion, we found that the endoscopic submucosal fibrosis of SSEN was closely associated with length of tumor, endoscopic depressed type, and timing of ESD from initial biopsy. The endoscopists need to apply extra caution when the length of tumor is over 20 mm, depressed, or over 21 days past after initial biopsy. Esophageal ESD for SSEN should be attempted immediately after biopsy.

## Study Highlights

### What is current knowledge

- Endoscopic submucosal dissection (ESD) is an effective treatment modality for superficial squamous esophageal neoplasia (SSEN)
- Submucosal fibrosis is an important obstacle to successful ESD

### What is new here

- Length of tumor greater than 20 mm and endoscopic depressed type are endoscopic predictive factors of submucosal fibrosis in SSEN
- To avoid submucosal fibrosis, ESD should be attempted immediately after biopsy for the diagnosis of SSEN

### Translational impact

- The endoscopists need to apply extra caution when the length of tumor is over 20 mm, depressed, or over 21 days past after Initial biopsy.
- Esophageal ESD for SSEN should be attempted immediately after biopsy.
